# Parkinson’s Disease: Conventional Pharmacotherapy, Drug Delivery Innovations, and Emerging Therapeutic Targets

**DOI:** 10.3390/brainsci16020226

**Published:** 2026-02-14

**Authors:** Deepika Raina, Chirag Marwah, Siddharth Singh, Ansab Akhtar

**Affiliations:** 1 School of Pharmacy, Graphic Era Hill University, Dehradun 248002, Uttrakhand, India; deepika709@gmail.com; 2 Chikara College of Pharmacy, Chitkara University, Rajpura, Patiala 140401, Punjab, India; chiragmarwah98@gmail.com; 3 Faculty of Pharmacy, School of Pharmaceuticals and Population Health Informatics, DIT University, Dehradun 248009, Uttrakhand, India; ss19970510@gmail.com; 4 Louisiana State University Health Sciences Center, Neuroscience Center, School of Medicine, New Orleans, LA 70112, USA

**Keywords:** Parkinson’s disease, dopamine, substantia nigra, dosage forms, novel targets, potential drugs

## Abstract

Parkinson’s disease (PD) is a progressive neurodegenerative disorder characterized by motor symptoms (bradykinesia, rigidity, resting tremor) and a wide range of non-motor features. The core pathological process is degeneration of dopaminergic neurons in the substantia nigra pars compacta, leading to striatal dopamine deficiency, while additional neurotransmitter systems contribute to non-motor symptoms. PD is a common age-related disorder; global estimates for 2019 indicate that more than 8.5 million people were living with PD, and prevalence increases steeply with age. Current pharmacological therapy is mainly symptomatic and is centered on levodopa and other dopaminergic strategies, but treatment response can be limited by motor fluctuations, dyskinesia, and adverse effects. Therefore, formulation and delivery innovations (e.g., dispersible preparations, intestinal gel, and continuous infusion approaches) aim to stabilize drug exposure and improve convenience, especially in patients with swallowing difficulties or advanced disease. This review summarizes conventional drug classes and their dosage forms, highlights formulation-driven strategies to improve efficacy and tolerability, and outlines emerging pathways and targets being explored for future therapies.

## 1. Introduction

Parkinson’s disease (PD) is a progressive neurodegenerative disorder that primarily affects dopaminergic neurons in the substantia nigra, leading to typical motor symptoms such as bradykinesia, rigidity, resting tremor, gait disturbance, and postural instability [1,2]. In addition, many patients experience non-motor symptoms (e.g., sleep disturbance, mood changes, cognitive symptoms, autonomic complaints, and fatigue), which reflect involvement of additional neurotransmitter systems and can overlap with other neurological conditions [2,3]. Although there is no curative therapy, available treatments can improve symptoms and quality of life. Current pharmacological management is mainly symptomatic and is complemented by rehabilitation approaches and, in selected cases, device-aided therapies [4].

Furthermore, oral levodopa undergoes extensive peripheral metabolism when used alone; therefore, it is typically co-administered with a peripheral dopa-decarboxylase inhibitor (e.g., carbidopa or benserazide) to increase the fraction available to cross the blood–brain barrier and to reduce peripheral adverse effects such as nausea. Patients who have swallowing difficulties or advanced motor fluctuations may benefit from formulation and delivery approaches such as dispersible preparations, intestinal gel infusion, and continuous infusion systems. At the same time, newer molecular targets are being explored for future therapies with better tolerability and potential disease-modifying effects. However, device-aided approaches may be invasive, and safety, tolerability, and feasibility remain important considerations.

The novelty of the current review revolves around the existing gaps in PD therapy in terms of safety issues and incomplete halting of disease progression with the conventional drugs. For example, the available formulations have disadvantages, including inadequate bioavailability or off-target delivery. To combat this drawback, subcutaneous infusion could provide better safety and efficacy. Furthermore, novel pathways with possible roles in the etiology and pathophysiology of PD, and those that have not yet been investigated thoroughly for PD, are discussed. These pathways could be the potential pharmacological targets for the concrete halting of disease progression, not merely for symptomatic relief, thereby being opposed to conventional medications. In addition, we have provided a list of drugs that could be repurposed based on PD pathophysiology. These ideas can be translated from preclinical to clinical aspects.

This review is structured to critically examine Parkinson’s disease management across three interconnected dimensions: limitations of conventional dopaminergic pharmacotherapy, formulation-based strategies aimed at improving pharmacokinetic stability and tolerability, and emerging molecular pathways that may hold future translational relevance. By integrating established therapies with dosage-form innovations and mechanistic insights into neurodegeneration, this review aims to bridge descriptive pharmacology with forward-looking therapeutic strategies, thereby extending beyond prior narrative overviews focused solely on symptomatic management.

### Methodology of the Review

A targeted literature search was performed in PubMed, Scopus, and Google Scholar up to September 2025 using combinations of terms such as “Parkinson’s disease”, “levodopa”, “dopamine agonist”, “drug delivery”, “intestinal gel”, “continuous infusion”, “deep brain stimulation”, “neuroinflammation”, and “ferroptosis”. Peer-reviewed articles in English (clinical trials, systematic reviews, and key preclinical studies) were prioritized. Reference lists of relevant papers were also screened to capture additional studies. The selected articles were synthesized narratively to summarize established therapies, formulation and delivery innovations, and emerging therapeutic targets.

## 2. Parkinsonism Classification

### 2.1. Idiopathic Parkinsonism

After Alzheimer’s disease, Parkinson’s disease is considered the second most common neurodegenerative disorder, wherein dopaminergic neurons in the substantia nigra pars compacta are affected. The motor symptoms get modulated by dopaminergic neurons, while the non-motor symptoms (NMSs) are concerned by peripheral as well as central non-dopaminergic neurons, including non-adrenergic, cholinergic, glutamatergic, and serotoninergic neurons. α-Synuclein aggregates from Lewy bodies and Lewy neurites in dopaminergic neurons, which are pathological hallmarks of PD [5,6]. Exposure to pesticides, associated melanoma, and a history of traumatic brain injury might enhance the risk of developing PD. A history of smoking, caffeine intake, and moderate to vigorous exercise might reduce the risk of PD [7]. Early PD is divided into three stages for the clinical approach: preclinical, prodromal/pre-motor, and clinical/motor stages [8]. Various motor symptoms in PD include resting tremors of the hand; slowing down of limb movements, reduced facial expression, abnormal handwriting, and reduced arm swing. Various non-motor symptoms in PD include constipation, anxiety, and cognitive impairment [9,10].

### 2.2. Vascular Parkinsonism

Vascular Parkinsonism (VP) is found in those patients who have vascular lesions in the brain parenchyma [11]. It is represented by lower-body Parkinsonism along with postural instability, absence of rest tremor, inadequate dopamine response, and the existence of corticospinal tract signs [12]. Dopamine substitutes MAO-B inhibitors, which can be useful for the treatment of Parkinsonism, but the efficacy of levodopa in VP is under discussion [13,14].

## 3. Epidemiology and Etiology

Parkinson’s disease is an age-related disorder. Global estimates for 2019 suggest that more than 8.5 million people were living with PD worldwide [15]. Prevalence rises markedly with age; a recent systematic review reported age-specific prevalence (per 100,000) of approximately 7 (40–49 years), 158 (50–59 years), 603 (60–69 years), 1251 (70–79 years), and 2181 (>80 years) [16].

The main reason behind Parkinson’s disease is not fully understood, but it is thought to reflect an interrelation between ageing, genetic susceptibility, and environmental factors [17]. As dopaminergic neurons degenerate, dopamine availability declines, and this contributes to clinical symptoms such as bradykinesia, rigidity, and difficulty in initiating movements [18].

When compounds like 1-methyl-4-phenyl-1,2,3,6-tetrahydropyridine (MPTP) are administered to humans or animals, they can lead to a form of Parkinsonism. MPTP acts as a powerful neurotoxin when it gets metabolized to 1-methyl-4-phenylpyridinium ion (MPP+) and causes suppression of mitochondrial complex 1 of the ETC (electron transport chain). This conversion ultimately results in the enhancement of reactive oxygen species (ROS), resulting in the death of neuronal cells [19]. Although levels of ROS are abundant in the substantia nigra pars compacta (SNpc) due to the degradation of dopamine by monoamine oxidase enzyme, the presence of antioxidative molecules like glutathione in SNpc reduces the damage caused by free radical reactions. In case of PD, this defense mechanism might get saturated or impaired [20].

Iron and copper, which are enriched in SNpc, are the required cofactors for the biosynthesis as well as the metabolism of dopamine. Thus, oxidative and reductive reactions of iron can also lead to the development of harmful metabolites and ROS. Various other factors, like toxicity due to nitric oxide, inflammation, mitochondrial dysfunction, and apoptosis, also contribute to the etiology of PD. However, PD due to genetic mutation is rare [21,22].

Oral intake of specific microbial metabolites like short-chain fatty acids causes neuroinflammation as well as motor symptoms. Various investigations prove that alterations in gut microbiota are linked to multiple clinical features of PD [23]. Dopamine causes hydrogen peroxide formation. When it attains a higher concentration, it accepts an electron from the Fe^2+^ (ferrous ion), which gets converted to a Fe^3+^ (ferric ion) and a hydroxyl free radical (OH-), leading to lipid peroxidation [24].

## 4. Pathophysiology

**Figure 1:**
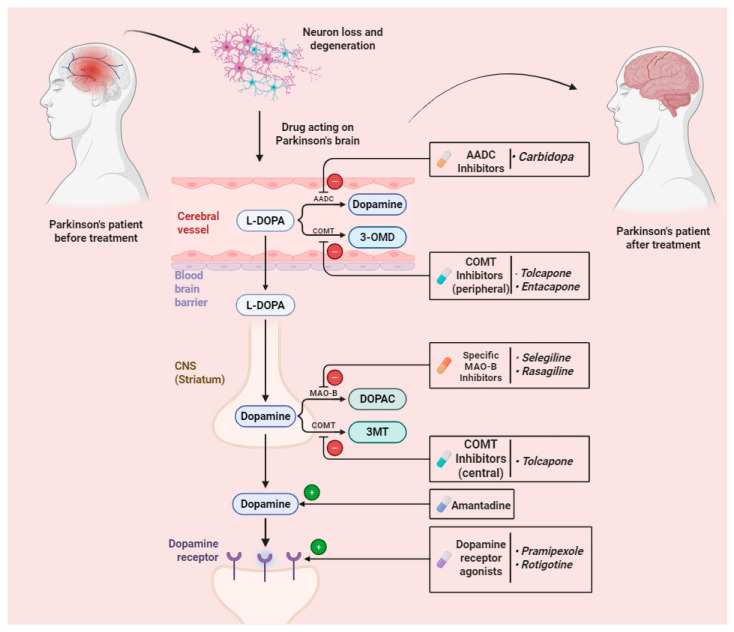
Drugs acting against Parkinson’s disease.

PD is mainly associated with the dopaminergic neurons’ degradation in the brain’s substantia nigra (Figure 1). This area is responsible for dopamine synthesis [25]. Although there is more information about PD pathophysiology that we get from daily updates, it is still primarily considered idiopathic (of unknown cause). It probably also includes host sensitivity and the interaction of environmental variables. There is a high proportion of cases that are idiopathic and fewer cases arising from an underlying genetic level [26].

Physiologically, PD-related symptoms stem from the reduction of several neurotransmitters, particularly dopamine. In Parkinsonism, the dopaminergic connection is lost with the neostriatum (putamen). This results in less inhibition and enhanced excitation from the subthalamic nucleus to the globus pallidum internal segment, and therefore inhibitory output to the thalamus and brainstem is increased [27].

### 4.1. Role of Dopamine

Dopamine is a neurotransmitter that transmits chemical signals across the synapse. Dopamine is released into the synapse by storage vesicles in the presynaptic neuron and then binds to receptors on the postsynaptic membrane, triggering dopaminergic signaling. Dopamine is cleared from the synapse mainly by reuptake and by enzymatic breakdown. Two key enzymes involved in its metabolism are monoamine oxidase (MAO) and catechol-O-methyl transferase (COMT). MAO inhibitors reduce dopamine breakdown and can prolong dopaminergic signaling, thereby improving motor symptoms in selected patients [28].

### 4.2. Progressive Loss of Dopamine

It is largely true that the reduced number of dopamine cells in PD subjects cannot be directly measured; however, measurements in neurologically healthy people show a slow, sudden fall in dopamine with age. The damage occurs at a much higher pace in Parkinson’s disease. Biochemical interventions and imaging studies explain that dopamine concentrations are significantly reduced as the motor symptoms are observed to arise [29].

### 4.3. Inflammation and Immune Response

Dopaminergic degeneration triggers appear to be multifunctional. Among these, inflammation and immune responses in PD contribute as significant dopaminergic degeneration mediators. Several community studies have proposed that people taking non-steroidal anti-inflammatory drugs (NSAIDs) have a reduced risk of developing idiopathic PD, explaining that anti-inflammatory drugs could be a favorable disease-transforming therapy for patients suffering from PD. Researchers use neuroimaging instruments to create an appropriate biomarker to test it in large-scale clinical imaging studies [30]. In this context, inflammation is widely recognized to have a robust association with the causation of PD pathology. Inflammatory cytokines like interleukins such as IL-6, IL-1, and TNF have been reported to be released upon the NF-B transcription factor. Either way, these cytokines are directly activated in the specific brain regions of the brain, like the substantia nigra, when it is regarded as neuroinflammation; otherwise, they have a peripheral origin when transported to the brain through the blood–brain barrier (BBB). Either way, this could be detrimental to the dopaminergic neurons of the brain and hence lead to neurodegeneration and PD pathology. Moreover, other inflammatory pathways, such as inflammasomes, MAPK, and C-reactive protein, also play a significant role in PD-related neuroinflammation. More specifically, the kynurenine pathway has been investigated and found to be dysfunctional in PD cases, which occurs due to impairment in tryptophan metabolism and neuroinflammation mediated by this pathway. Additionally, gut-brain axis-associated neuroinflammation in PD could be another etiology arising from dysbiosis in gut microbial flora. This could be due to a weaker immune system, improper diet, or aging. Lastly, the immune system-related microglial activation comes into play upon alpha-synuclein deposition, oxidative stress, and mitochondrial dysfunction. This creates a vicious cycle in the progression of PD pathology and hence in the appearance of symptoms [31,32].

## 5. Conventional Treatment of Parkinson’s Disease by Pharmacological Drugs and Their Dosage Forms

Numerous drug molecules and dosage forms have been reported to be currently used for treating Parkinson’s disease (Figure 1).

### 5.1. Levodopa

Basal ganglion degeneration in the brain, as seen in PD, primarily affects dopaminergic neurons, leading to dopamine deficiency. Levodopa is absorbed by other dopaminergic neurons from where it undergoes decarboxylation to form dopamine in the presynaptic terminal. Levodopa is commonly paired with benserazide or carbidopa. They do not cross the BBB, but by inhibiting the aromatic acid decarboxylase enzyme that catalyzes this reaction, the peripheral conversion of levodopa into dopamine is blocked. Therefore, dopaminergic adverse effects are decreased, core supply is enhanced, and hence L-dopa dosage can be lowered [33].

In addition to this approach, continuous intestinal infusion of levodopa gel (Duodopa (AbbVie Limited)—a combination of levodopa with carbidopa) is more efficient in reducing severe motor changes compared to conventional levodopa. This is basically due to a more appropriate intake of levodopa. The remaining dopaminergic neurons absorb levodopa and transform it into dopamine, which helps to somewhat make up for the dopamine shortage that results from the loss of neurons in the substantia nigra pars compacta. Some of the advantages of levodopa include the improvement of the quality of life of patients, wider availability, and long-lasting symptomatic relief. However, for extensive use, this therapy is presently prohibitively costly [34,35]. Additional disadvantages of levodopa are motor symptom fluctuations, a larger number of adverse events, including gastrointestinal and psychiatric complications, etc. Adjusting the dosage regimen, reducing the influence of food, and starting with a lower dose are some of the strategies that can minimize the complications associated with levodopa [36]. Scientists continue to concentrate on developing other long-acting oral preparations and other drug delivery methods that might enable enhanced clinical efficacy and fewer side effects in the future.

#### 5.1.1. Available Dosage Forms of Levodopa

##### Tablets

It comes in different strengths, depending on the dosage prescribed by the physician. Controlled and/or prolonged release capsules or tablets help levodopa to enter the body steadily instead of a sudden spike. The adverse effect of consuming high doses of levodopa may include involuntary movements (dyskinesia). Often, controlled release methods may reduce the number or frequency of dyskinesia, and they can be taken at night to reduce stiffness [33]. Levodopa tablets possess dosage flexibility, allowing adjustment of the required dose by splitting them according to the needs of the patient’s demographic characteristics and symptom severity. However, dysphagia might result in motor symptoms fluctuating [37]. The bitter taste of the tablets may also limit their use.

##### Dispersible Tablets

Dispersible tablets can be consumed by mixing them with water. This works faster than capsules, because the active ingredient does not need to be disintegrated in the stomach. It can also be used when an individual is having difficulty swallowing tablets or capsules. The non-dispersible tablet and capsule dosage forms should not be crushed or dissolved in liquid when prescribed by a prescriber [33]. Dispersible tablets are more appropriate than normal tablets for critically ill or very elderly patients, and even for pediatric patients who rarely encounter Parkinsonism. Due to no requirement for water, this formulation can be administered to patients when there is no or limited access to water. Despite that, durability during packaging and shipping is one of the drawbacks associated with this category of tablets [38].

##### Intestinal Gel

In this type of dosage form, the drug is pumped continuously into the intestine with the help of a surgical tube. When these intestinal gels are prescribed to patients, they are less likely to experience involuntary movements. It may aid in controlling symptoms at night [33]. A systematic review on levodopa–carbidopa intestinal gel has demonstrated that it alleviates treatment-resistant gait-freezing behavior of PD patients, which has constantly been a challenge with oral levodopa. This formulation has overcome the problem of sustainability and steadiness of the conventional formulations, making it a promising dosage form for a specific type of PD symptom. Still, additional research is required to confirm the reliability of intestinal gel [39,40].

In advanced disease, continuous delivery approaches have been developed to reduce plasma level fluctuations and motor OFF time. These include intestinal gel infusion and subcutaneous infusion systems that provide a more stable levodopa exposure using a pump-based device.

A randomized double-blind Phase 3 clinical trial published in The Lancet found that the subcutaneous levodopa–carbidopa (ND0612) treatment was more efficacious and safer with fewer motor fluctuations, such as troublesome dyskinesia in PD patients, compared to orally administered formulations. This study signifies that subcutaneous infusion of levodopa over 24 h maximizes the tolerability and minimizes the chances of adverse effects and drug interactions, with further diminished need for surgical interventions [41]. Despite all these benefits, the requirement of a pump and itching at the site of infusion are some of the complications that cannot be ignored while deciding on a proper drug delivery system.

### 5.2. Dopamine Agonist

Dopamine receptor agonists were released into the market in 1978 to treat PD. The frequently used agonists contain an ethanolamine moiety and can be classified as ergot and non-ergot-derived based on receptor specificities [42]. These drugs enhance the therapeutic efficacy of the dopamine system by binding to dopaminergic receptors and do not require transformation into dopamine, unlike levodopa [43]. Rotigotine is a dopamine agonist available as a skin patch (transdermal patch) for the treatment of PD. This can be effective when a person is having difficulty swallowing tablets or experiencing gastritis difficulties. It may lower stomach upsets. This patch is to be kept in place for 30 s. It lasts 24 h once in place. The patch might cause a mild skin reaction, like reddening or itching. However, it is often mild or moderate and affects only the area where the patch was placed and will disappear eventually after a couple of hours when the patch is removed. Every day, the patch should be moved to a different place to prevent irritation. The patch must be kept in a refrigerator [43]. Likewise, in case of “off episodes” of PD, meaning that when other medication effects wear off, causing worsening of PD symptoms, an injectable dopamine agonist, namely apomorphine, can be used.

Generally, levodopa and carbidopa are the drugs used for the treatment of PD at the current time. As earlier mentioned, their basic mechanism of action includes the conversion of levodopa to dopamine by an enzyme called DOPA, which is naturally occurring. This happens at both the peripheral circulation and even the Central Nervous System (CNS), after levodopa has crossed the blood-brain barrier. When the central dopamine receptor gets activated, it tends to reduce the symptoms of PD, whereas when the peripheral dopamine receptor gets activated, it results in nausea and vomiting. Due to this, levodopa is generally administered with a DOPA decarboxylase inhibitor. Here, carbidopa is used, which is hydrophilic and is unable to cross the BBB. However, it prevents the peripheral conversion of levodopa to dopamine and reduces its peripheral side effects. Thus, with the co-administration of carbidopa, there is an increase in the amount of levodopa in the bloodstream, which is free to enter the brain. Carbidopa inhibits levodopa’s peripheral dopamine metabolism, so that a large quantity of levodopa is accessible for transport into the central nervous system through the BBB. By antagonizing the external disintegration of levodopa to dopamine, it reduces dopamine-producing side effects like vomiting and nausea. In addition, dopamine receptors are directly activated by synthetic dopamine agonists [43,44].

### 5.3. COMT Inhibitors

Dopamine is a chemical messenger. PD symptoms are produced by a decrease in dopamine concentrations because of the death of the brain’s nerve cells that help in producing dopamine. Catechol-O-methyl transferase (COMT) is an enzyme that breaks down dopamine present in the brain [44]. Thus, blocking COMT’s action is one of the ways to treat PD. COMT inhibitors block the enzyme COMT and decrease the metabolism of levodopa and dopamine, either at the periphery or in the central part of the brain, for effective treatment of PD. There are two primary COMT inhibitors: entacapone and tolcapone. Along with levodopa, these medications are used as adjunct therapy, as they function more effectively when not used alone. They may be used when the levodopa dose does not work long enough or if it wears off. These drugs increase “on time” and decrease “off time”, thereby facilitating the levodopa effect and delaying the return of PD symptoms, respectively [45]. COMT inhibitors may also be used when elevated doses of levodopa cause severe side effects. COMT inhibitors are given in tablet form. They are also available in combination tablets that consist of levodopa, carbidopa, and entacapone, a COMT inhibitor [46]. COMT inhibitors, especially tolcapone, might result in liver dysfunction and gastrointestinal disturbances. On the other hand, entacapone often causes urine discoloration.

### 5.4. MAO-B (Monoamine Oxidase B) Inhibitors

Monoamine oxidase-B inhibitors (MAO-B) can help the nerve cells use dopamine. This is achieved by inhibiting the MAO-B enzyme, which targets the brain’s dopamine. The MAO-B inhibitors block the breakdown of dopamine in the CNS. This helps increase the quantity of dopamine, which can be used by the brain, helping to ease PD symptoms. The three main MAO-B inhibitors that have been recently developed are rasagiline, selegiline, and safinamide. Some studies have delineated that these drugs also have the potential to weaken the disease progression, not merely provide symptomatic relief, in addition to their approved indications [47,48]. However, there is still a lack of definite disease-modifying evidence. These drugs are available in tablet form. There is also another dosage form available that dissolves on the lingual region (i.e., tongue). It is used when the individual has difficulty swallowing [49].

### 5.5. Amantadine

Amantadine is an antiviral drug that has been repurposed as an antidyskinetic agent, often used alone or in combination with levodopa. Its action against sudden uncontrolled movements associated with PD is due to its agonist action on dopamine receptors. Occasionally, it is also used to treat tremors and stiff muscles. However, this drug is not commonly used or prescribed by physicians due to its low efficacy and psychiatric adverse effects. Amantadine is usually given in combination with levodopa for treating PD. This drug is available in capsules as well as syrup [50].

### 5.6. Anticholinergic Medication

Anticholinergic drugs prevent the effect of acetylcholine (Ach), which is a neurotransmitter. ACh sends messages to muscles from nerves. The amount of dopamine and acetylcholine activity in the brain is maintained. However, in PD, a dopamine-deficient brain leads to acetylcholine being overactive. Anticholinergics help restore this equilibrium by preventing the action of acetylcholine and helping to decrease the symptoms of PD, particularly tremors [51]. Some common examples of anticholinergics include benztropine, procyclidine, diphenhydramine, and trihexyphenidyl (Table 1). As acetylcholine is one of the most important neurotransmitters in the brain, anticholinergic drugs elicit excessive side effects, including dry mouth, constipation, blurred vision, and increased heart rate. Hence, their use is restricted in patients who cannot tolerate them. Notably, anticholinergics’ acetylcholine-depleting effect has been revealed to lead to possible dementia in elderly individuals taking the drugs of this classification. This is due to the role of acetylcholine in synaptic plasticity and memory formation, thereby discouraging the prescription of anticholinergic drugs for PD [51].

## 6. Clinical Positioning of Established Therapies (Overview)

Current management emphasizes individualized, symptom-driven therapy rather than rigid age-based algorithms. Levodopa remains the most effective treatment for motor symptoms and may be initiated at any disease stage when functional impairment is present. Evidence from randomized trials demonstrates that dyskinesia risk relates more strongly to disease duration and cumulative exposure than to early initiation.

Contemporary management of Parkinson’s disease emphasizes individualized, symptom-driven treatment rather than rigid age-based algorithms. Levodopa remains the most effective therapy for motor symptoms and may be initiated at any disease stage when functional impairment warrants treatment. Evidence from randomized trials indicates that levodopa-induced dyskinesia is more closely related to disease duration, cumulative exposure, and pulsatile dopaminergic stimulation than to early initiation alone. Dopamine agonists, MAO-B inhibitors, and COMT inhibitors are used as adjuncts based on symptom profile, cognitive status, comorbidities, and patient preference. Current guidelines prioritize quality of life, early symptom control, and proactive monitoring of motor and non-motor complications over attempts to delay levodopa use.

Treatment selection is individualized based on age, symptom burden, functional impact, comorbidities, and adverse-effect risk. Levodopa remains the most effective option for motor symptoms and is commonly introduced when symptoms affect daily activities. In selected patients with early disease, MAO-B inhibitors or dopamine agonists may be considered, either alone or as add-on therapy, to reduce OFF time and delay wearing-off. In advanced disease, adjunct therapies (COMT inhibitors, MAO-B inhibitors, amantadine) and device-aided approaches can be considered to manage motor fluctuations and dyskinesia. Non-motor symptoms should be screened routinely and managed with evidence-based interventions as summarized in Table 1.

## 7. Advanced Treatment Strategies

The approaches below represent adjunct or emerging strategies that have been evaluated in clinical or preclinical studies with variable outcomes. They should be interpreted as investigational or context-specific options rather than routine first-line therapy.

### 7.1. Inosine

Inosine is a purine nucleoside that has a variety of intracellular functions and delivers an extracellular modulatory signal. It has an anti-inflammatory effect on neurons and promotes cellular growth. The protective consequences of inosine treatment may be mediated by its metabolite urate, which has strong antioxidant properties and has demonstrated protective effects in preclinical PD studies, as well as the potential to retard the development and progression of PD [52]. Urate is a significant antioxidant that circulates in the human body, and it has an inverse association with PD risk [53]. Oxidative stress is found to be a pathophysiological mechanism in the development of PD [54]. Clinical studies have shown that urate levels in serum or cerebrospinal fluid (CSF) correlate with a decreased risk of developing PD in healthy populations and with a decreased risk of clinical progression in patients suffering from PD. Notably, in the past, a clinical trial, namely the SURE-PD3 randomized placebo-controlled clinical trial, found that inosine was safe and significantly increased urate levels in blood and CSF; however, the trial was later halted due to its inability to slow down the progressive nature of PD. This discontinuation was necessary as the main purpose of newer clinical investigations focuses on the cure or attenuating the disease advancement rather than only providing symptomatic relief, which is already achieved by conventional drugs. Despite this failure, the study provided notable information regarding its tolerability, efficacy, and urate bioavailability, paving the way for future direction in the relevant investigations [55]. Apart from this, in cellular and animal models of PD, it is found that elevation of urate levels reduces oxidative stress and loss of dopaminergic neurons [56,57,58,59,60]. Inosine, when administered orally, causes a rapid increase in serum urate, which helps in treating PD [61].

### 7.2. Safinamide

The major drawback of dopaminergic drugs is that they don’t reduce motor side effects completely while treating PD. Unmet requirements can be achieved by the advancement of an adjunctive drug for dopamine replacement. Safinamide (Xadago) is an anticonvulsant drug that has possible therapeutic effects and can be beneficial in the symptomatic relief of neurodegenerative diseases, like PD [62]. Safinamide is a selective MAO-B inhibitor that is orally absorbed with a half-life of about 24 h. Its significant advantage is that its bioavailability is very high (>90%) and attains steady-state plasma levels within a week [63]. Safinamide is a small water-soluble molecule with two main defined mechanisms of action—it inhibits dopamine breakdown by inhibiting the MAO-B enzyme modulation of sodium and calcium channels and inhibiting glutamate [64]. Safinamide inhibits MAO-B reversibly, which reduces the dopamine reuptake at synaptic junctions. This enhances the availability of dopamine at the synaptic junction as a neurotransmitter. Besides this, safinamide has some non-dopaminergic mechanisms of action; for example, it blocks site 2 of voltage-gated N-type sodium channels and calcium channels while they are in their inactivated state, which acts to inhibit the release of glutamate. Since L-type calcium channels are not affected by safinamide, it does not have any cardiovascular effects [65]. Safinamide does not affect the COMT enzyme [66]. It acts on voltage-gated sodium channels and decreases glutamate release, which leads to the activation of its symptomatic potential.

### 7.3. Coenzyme Q10

Many previous studies have shown that patients suffering from PD have decreased complex I activity in the postmortem substantia nigra by 30–40%. Various preclinical and clinical investigations have been performed to find a drug that can successfully reduce the progression of PD. Although various PD genes whose function is associated with mitochondrial function have been discovered, none of them show strong therapeutic effects. Coenzyme Q10 is the most powerful antioxidant, which acts as an electron acceptor for complexes I and II, and this can decrease free radical generation. CoQ10 shows neuroprotective potential in several in vitro and in vivo neuronal toxicity studies. It has been found that, upon oral administration, coenzyme Q10 can decrease the reduction of dopaminergic axons and dopamine in the striatum of the brain [67,68]. Currently, clinical studies illustrate that 300 mg/day of CoQ10 can significantly attenuate PD symptoms, which has led to an increased interest in nutritional supplements of coenzyme Q10 for neurodegenerative disease. It is an essential cofactor and is also a potent antioxidant involved in mitochondrial oxidative phosphorylation [69]. A Phase 3 randomized, placebo-controlled, double-blind clinical study in early PD patients found that coenzyme Q10 in different doses was safe and tolerable; however, the clinical outcome in terms of minimizing disease progression and demonstrating observable therapeutic efficacy was not significant enough to continue the study further [70].

### 7.4. Laminin-511

Parkinson’s disease is an extrapyramidal motor disorder resulting from the loss of dopaminergic neurons present in the substantia nigra (located in the ventral midbrain). Nowadays, treatment strategies are developed for patients with PD, mainly targeting the associated symptoms; therefore, it is necessary to determine a mechanism that can block the loss of midbrain dopaminergic neurons. However, there are some molecules known as extracellular matrix (ECM) molecules, about which little information is known regarding their function in specific midbrain dopaminergic neurons. Laminin is a family of extracellular matrix proteins that has gained importance in recent years because it has the potential to control not only adhesion, but also stem cell survival, maintenance, migration, and differentiation. Laminins are large trimeric proteins that consist of three chains (a1–5, b1–3, and g1–3). Different data indicate that in the extracellular space, LM511 is present, which is a strong signal of survival and differentiation for dopaminergic midbrain neurons. LM511 signals were found to activate Yes-associated protein 1 (YAP) via the integrin a3b1 (a transcriptional regulator and central component of the Hippo pathway). In addition, the growth of axons and cone formation have previously been shown to be a requirement for midbrain DA neurons to function adequately, and it is necessary for the clinical application of dopamine neurons, which are derived from stem cells [71,72,73]. Interestingly, LM511 has been tried to be incorporated in the growth of stem cells like iPSCs, strengthening its clinical importance in the replacement therapy for PD patients. In this regard, several studies have demonstrated the utilization of perlecan-conjugated laminin 511/521-E8 fragments to depict enhanced maturation of grafted dopamine progenitors in preclinical models, which further prompted the initiation of clinical trials. For example, currently, a Phase 1 clinical trial is investigating patients’ own blood-derived autologous iPSC-derived dopamine neurons in PD subjects. This approach has the potential to replace the neurodegenerative dopamine neurons with healthier ones, thereby creating a way to slow down the disease progression [74].

### 7.5. Glial Cell Line-Derived Neurotrophic Factor (GDNF)

Neurotrophic factor (GDNF) derived from glial cells has powerful neurotrophic effects, not exclusively on dopaminergic neurons; both neuroprotective and neurogenerative effects are consistently demonstrated in animal models in the form of a viral vector or continuous infusion in the cerebral ventricle (ICV). In clinical trials, GDNF was given monthly intracerebroventricular bolus injections; however, there was no effect due to less penetration into the brain’s target areas, and there were severe side effects [75]. The result was related to a significant increase in putamen 18F-dopa uptake on positron emission tomography (PET), and increased tyrosine hydroxylase immunopositivity nerve fibers are found in the infused putamen [76]. Early-stage clinical trials of GDNF have produced more promising results than the later ones in demonstrating clinical benefits like improving motor symptoms because of elevated dopamine uptake in the brain associated with PD pathology [77]. However, further trials are required to optimize its delivery and dosage.

### 7.6. Rytary

Rytary is an extended-release formulation of carbidopa and levodopa, used in the treatment of early, moderate, and advanced stages of PD. The medication was approved by the US-FDA in 2015. Rytary is a singular formulation of carbidopa and levodopa, offering a longer and more efficient method of action than normal combinations of carbidopa and levodopa. Rytary capsules are formulated so that they contain beads to produce carbidopa and levodopa at varying speeds when absorbed into the stomach. This allows for prolonged absorption. Carbidopa prevents the breakdown of levodopa until it enters the brain. After entering the brain’s systemic region, levodopa is converted into dopamine by nerve cells to replenish the brain’s supply and to treat Parkinson’s disease [78,79].

### 7.7. Deep Brain Stimulation [DBS]

DBS is a neurosurgical technique that is currently being utilized extensively in the care of severe PD, notably in patients who have drug-resistant motor fluctuations and dyskinesias after having their pharmacotherapy improved. The procedure involves the stereotactic implantation of electrodes in specific deep brain nuclei, mainly the subthalamic nucleus (STN) and internal segment of the globus pallidus (GPi). They are connected to an implantable pulse generator, which delivers regulated electrical currents to re-establish normal brain circuitry by controlling abnormal electrophysiological activity [80,81].

STN stimulation has been associated with a substantial reduction in dopaminergic medication requirements, thus evading levodopa-induced dyskinesia complications. GPi targeting, however, is preferentially used in clinical situations involving dyskinesias because direct pallidal stimulation adequately suppresses these involuntary motor manifestations. Both anatomical targets yield substantial motor benefits; however, STN has been more frequently associated with side effects of the neuropsychiatric and cognitive type. Stricter patient selection, therefore, with input from rigorous preoperative neuropsychological evaluation, remains part of the assessment process for DBS candidacy [80,81,82].

Clinically, DBS has been demonstrated to be successful in sustaining benefit for motor symptom relief, as indicated by large improvements in Unified Parkinson’s Disease Rating Scale (UPDRS) scores, reduction in “off” times, and improvements in health-related quality of life. Additionally, its pharmacological burden reduction provides benefit in the form of limiting the development of drug-induced motor complications [83,84].

Although antioxidant, neurotrophic, and mitochondrial-targeted strategies have demonstrated robust neuroprotective effects in preclinical models, translation into clinically meaningful disease modification has remained elusive. Large, randomized trials investigating agents such as inosine, coenzyme Q10, and GDNF failed to confirm sustained clinical benefit, underscoring the complexity of Parkinson’s disease pathophysiology and the challenges of therapeutic translation.

## 8. Novel Pathways in Parkinson’s Disease

### 8.1. Pyroptosis and Neuroinflammation in Parkinson’s Disease

A study by D’Souza and his colleagues reported the term “pyroptosis”, in which “pyro” means “fever” and “ptosis” means “falling”. Pyroptosis is a smart inflammatory programmed cell death that includes canonical and non-canonical mechanisms moderated by caspase-1, 4, 5, and 11 [85]. This caspase-mediated cell death in the substantia nigra can trigger dopaminergic neuronal death, leading to neurodegeneration. Evidence supporting a role for pyroptosis in Parkinson’s disease is currently derived predominantly from preclinical studies, including cellular and animal models of dopaminergic neurodegeneration. Furthermore, mitochondrial dysfunction and excessive production of ROS due to a lack of oxygen supply are strongly responsible for the activation of NLRP3 inflammasomes within microglia and neurons. NLRP3 has been reported to cause inflammation in humans. Initially, low expression of the NLRP3 protein cannot stimulate the activation process; however, TLR agonists activate the NF-κβ pathway, which further enhances NLRP3 expression. NLRP3 can recruit and cleave pro-caspase-1 and form caspase-1. The activation of Caspase-1 produces interleukins-1β and -18 and is responsible for GSDM (Gasdermin D) cleaving. The N-terminal of GSDM causes pore formation in the plasma membrane. This pore formation becomes responsible for cell swelling and lysis, followed by inflammatory cytokines, and causes pyroptosis [86,87]. At present, direct clinical evidence linking pyroptosis to disease progression in human PD remains limited. Although elevated inflammasome-related markers and inflammatory cytokines have been detected in postmortem brain tissue and peripheral samples from PD patients, these findings are associative and do not establish causality. Importantly, no pyroptosis-targeting therapies have yet advanced to late-stage clinical trials in PD. The importance of pyroptosis as a potential therapeutic target is evolving owing to its association with GSDMD and NLRP3 inflammasome pathways. This phenomenon of pyroptosis, in conjunction with neuroinflammation, is highly susceptible to producing PD-related pathology. The pro-inflammatory cytokines elevated from these pathways play a key role in PD-related neuroinflammation and dopaminergic nigrostriatal neurodegeneration, and hence, stopping or weakening the same could be crucial for the treatment of PD. Nevertheless, translational efforts are emerging, particularly in the identification of circulating pyroptosis-associated proteins and non-coding RNAs as potential biomarkers. These approaches remain exploratory but may aid in early disease stratification rather than immediate therapeutic intervention. Overall, pyroptosis should currently be regarded as a mechanistic and biomarker-oriented research target, rather than a clinically actionable therapeutic pathway [88].

### 8.2. PINK1 (PTEN Induced-Putative Kinase 1) and Parkinson’s Disease

In 2001, PINK1 was identified, and in 2004, its role in early-onset PD was discovered. Several studies have described the working relationship between PINK1 and parkin for the elimination of unhealthy mitochondria. The pathogenic relevance of PINK1 is strongly supported by genetic and preclinical evidence, particularly in early-onset familial Parkinson’s disease. Mutations in PINK1 lead to mitochondrial dysfunction. As a result, endosomal ATP deposition and ROS formation occur, which are responsible for oxidative stress. In addition, the deficiency of PINK1 creates a hindrance in respiratory chain function and enhances α-synuclein aggregation and apoptosis susceptibility in neuronal cells. This leads to the basic pathophysiology related to PD [89,90,91,92]. From a clinical perspective, PINK1 mutations account for a small proportion of PD cases, and their relevance to sporadic late-onset PD remains uncertain. While mitochondrial dysfunction is a well-recognized feature of PD, direct clinical modulation of the PINK1–parkin pathway has not yet been achieved. Current therapeutic strategies, including gene editing and small-molecule activators of mitophagy, remain at an early experimental stage. PINK1 action in complement to parkin protein has an additional function in the survival of mitochondrial energy, and hence its dysregulation has been reported to possess the potential to cause PD-related motor abnormalities. Interestingly, mutations in PINK1 or parkin lead to the familial type of PD, as a mutated PINK1/parkin is not as efficient in clearing the dysfunctional mitochondria responsible for oxidative stress. Hence, genetic manipulation or editing through CRISPR/Cas9 could be a good target for early-onset PD. In this regard, targeting PINK1 could serve a dual purpose of correcting oxidative stress and mitochondrial dysfunction associated with PD-related neurodegeneration. The small molecules to upregulate this pathway are currently under investigation [93].

Thus, PINK1 represents a biologically validated preclinical target, with ongoing translational research focused on pathway modulation and biomarker development. However, clinical applicability beyond rare familial PD cases has yet to be established, and therapeutic readiness should be interpreted cautiously.

### 8.3. Ferroptosis and Parkinson’s Disease

Iron accumulation and lipid peroxidation lead to cell death known as ferroptosis. Preclinical studies provide strong mechanistic evidence for ferroptosis involvement in PD, particularly through excessive iron accumulation in the substantia nigra, which is responsible for catalyzing the Fenton reaction that produces ROS. This ROS formation causes lipid peroxidation, which damages the cell membrane. In a normal brain, GPX4 enzymes encode for glutathione peroxidase, which protects the brain. However, in PD patients, inhibition of GPX4 results in downregulation of glutathione peroxidase enzyme and glutathione depletion. As a result, mitochondrial shrinking and cell membrane damage arise, causing cell death via ferroptosis [94,95,96]. In this context, PD has one of its etiological factors as oxidative stress arising from ferroptosis, which appears to be a worthwhile investigational target. Likewise, glial cells also get destroyed up to the appearance of ferroptosis. This distorts the supportive network and protective aspect of the neuronal cells in the substantia nigra, leading to PD-related pathology. Hence, in terms of therapeutic strategies, antioxidants, glial cell regulators, iron chelators, PKC inhibitors, and ferroptosis inhibitors such as Lip-1 or Fer-1 can be further investigated to depict their role in inhibiting ferroptosis. In addition, glutathione system modulators have also been reported to regulate the ferroptosis phenomenon associated with PD [97]. In animal and cellular PD models, ferroptosis inhibitors, iron chelators, and antioxidants have demonstrated neuroprotective effects. However, clinical evidence directly implicating ferroptosis as a driver of disease progression in human PD remains indirect. While elevated brain iron levels have been detected using neuroimaging techniques, no ferroptosis-specific therapeutic agents have yet been validated in clinical trials for PD.

Consequently, ferroptosis currently represents a promising mechanistic framework for understanding oxidative neurodegeneration, with translational relevance focused on biomarker discovery and repurposing of iron-modulating agents. Its therapeutic potential remains preclinical to early translational.

### 8.4. Zona Incerta (ZI) and Parkinson’s Disease

The zona incerta (ZI), a subcortical brain region adjacent to the subthalamic nucleus (STN), is becoming increasingly known for its therapeutic targetability in PD. Unlike other pathways discussed in this section, ZI involvement in PD has limited but tangible clinical relevance, primarily through deep brain stimulation (DBS) studies.

Clinical and preclinical evidence indicate that DBS of the ZI, especially the caudal segment, has the potential to significantly alleviate cardinal motor symptoms, including tremor, muscle stiffness, and bradykinesia. In contrast to the STN, a popular target of DBS, the ZI has diffuse connections with brain structures involved in the control of movement, such as the basal ganglia, thalamus, motor cortex, brainstem, and cerebellum. This distributed connectivity may explain its capacity to regulate a variety of motor circuits [98,99].

Research by Londei et al. has shown that the ZI is a highly connected neural hub. They found that most ZI neurons actively communicate with brain areas involved in movement, emotion, and sensation using high-resolution mouse recordings. According to the results, one-third of ZI neurons had functional connections with multiple brain areas. Some neurons communicated with nearly all recorded regions simultaneously. Loop-like assemblies, where impulses loop from the ZI to another location and back to other ZI neurons, were found. This “looped” signal presumably allows feedback integration, which optimizes motor responses and adapts to environmental demands. These loop-like patterns were most common in neurons that were highly connected to the rest of the brain, supporting the idea that the ZI coordinates and organizes brain activity. These traits make it a good candidate for PD treatments like DBS [100].

After damaging the dopamine neurons in a PD model animal, researchers found that the number of GABA-releasing neurons in the ZI was significantly reduced. Scientists used advanced methods to specifically stimulate the GABAergic neurons in the ZI to investigate their therapeutic potential. Single and repeated stimulation of the neurons enhanced movement and balance in PD mice. Motor function was not only enhanced by repeated stimulation, but also by elevated levels of dopamine in the striatum, the brain area severely impacted in PD. The ZI is a focal point, a pivotal area determining motor symptoms in PD, and holds promise for being the basis of a future therapy in which ZI GABAergic neurons are stimulated, possibly to provide both relief from symptoms and neurochemical benefit [101]. Therefore, ZI neuromodulation should be considered a translational and early-clinical research avenue, rather than an established therapeutic strategy.

Overall, mechanisms such as pyroptosis, ferroptosis, PINK1-mediated mitophagy, and zona incerta neuromodulation remain largely preclinical. At present, these pathways should be regarded as investigational rather than clinically actionable.

In an animal model of PD, dopaminergic neuron injury was associated with a reduction in GABA-releasing neurons in the ZI. Targeted stimulation of ZI GABAergic neurons improved movement and balance in PD mice, and was accompanied by changes in striatal dopamine levels [101]. These findings suggest that ZI circuits can modulate motor networks that are affected in PD; however, the evidence is preclinical, and this does not replace the central role of nigrostriatal dopaminergic degeneration in human disease. Further clinical studies are required to define the translational relevance.

## 9. Discussion

Current pharmacotherapy for PD remains largely symptomatic. Levodopa and related dopaminergic strategies provide the strongest motor benefit, but long-term treatment is commonly complicated by wearing-off, motor fluctuations, and dyskinesia. These limitations reflect, in part, the short half-life of levodopa and fluctuating plasma exposure with conventional oral dosing, as well as patient-related factors such as delayed gastric emptying and variable absorption.

Formulation and drug-delivery innovations attempt to address these practical issues by improving convenience, supporting more predictable exposure, and reducing peak–trough variability. Dispersible preparations can assist patients with swallowing difficulties; intestinal gel and pump-based continuous infusion approaches aim to provide more stable levodopa delivery in selected patients with advanced disease. However, device-aided approaches may be invasive and require careful patient selection, monitoring, and management of procedure-related adverse events.

Beyond dopaminergic replacement, there is growing interest in pathways linked to mitochondrial dysfunction, oxidative stress, and neuroinflammation. Targets such as PINK1-related mitochondrial quality control, ferroptosis, and inflammasome-associated inflammatory signaling are being explored in preclinical models, but clinical translation will depend on robust biomarkers, well-defined patient subgroups, and meaningful outcomes. Overall, a pragmatic near-term direction is to combine optimized symptomatic therapy with delivery improvements, while continuing carefully designed clinical studies of candidate disease-modifying approaches.

## 10. Patent Information

Table 2 summarizes important patents in PD, focusing on efforts to improve treatment effectiveness and patient care.

## 11. Conclusions

PD is a progressive and multifactorial neurodegenerative disease that is defined by the degeneration of dopaminergic neurons. There are several current pharmacotherapies, mainly focused on dopaminergic replacement and providing symptomatic control. However, they are not able to reverse disease progression. Existing therapies also come up with several complications, including unwanted side effects and inadequate efficacy. In this review, we have elaborated on the conventional therapies and dosage forms, highlighting their advantages and limitations, such as their pharmacokinetic issues and off-target activity. Contextually, we added a few novel potential therapeutic strategies that can be investigated preclinically and clinically. Some of these include subcutaneous infusion devices, adjunct neuroprotective drugs such as inosine and coenzyme Q10. We also addressed new molecular targets such as ferroptosis, pyroptosis, PINK1, and mitochondrial pathology. The future of PD management must shift toward multi-targeted therapeutic approaches that serve neuroprotection, targeted delivery, and minimized toxicity with the help of drug delivery systems. Progress in nanotechnology, gene editing, and stem cell-derived dopaminergic neuron transplantation for disease treatment represents advanced directions for PD research. Moreover, harnessing artificial intelligence in early diagnosis and therapeutic screening can transform the clinical landscape of PD in the immediate future.

Despite major advances in symptomatic therapy and drug delivery, no current intervention prevents disease progression. Future progress will depend on integrated strategies combining optimized delivery systems, mechanistic insights, biomarker development, and individualized patient care.

PD is a progressive and multifactorial neurodegenerative disease characterized by degeneration of dopaminergic neurons and a broad range of motor and non-motor symptoms. Current pharmacotherapies are mainly symptomatic and are centered on levodopa and other dopaminergic strategies; however, long-term use can be limited by wearing-off, dyskinesia, and adverse effects. Formulation and delivery innovations, including dispersible preparations, intestinal gel, and continuous infusion approaches, aim to improve the predictability of exposure and clinical convenience in selected patients. In parallel, emerging molecular targets linked to mitochondrial dysfunction, oxidative stress, and neuroinflammation are being investigated, but most remain preclinical and require stronger clinical validation. Future progress is likely to come from combining optimized symptomatic regimens with patient-friendly delivery systems and well-designed studies of disease-modifying strategies.

## Figures and Tables

**Table 1:** Summary of therapeutic strategies in Parkinson’s disease.

Category/Therapy	Examples	Mechanism of Action	Clinical Role/Remarks
First-line dopaminergic therapy	Levodopa/carbidopa	Levodopa is a dopamine precursor that crosses the BBB; carbidopa inhibits peripheral decarboxylation, increasing CNS availability.	Most effective therapy for motor symptoms; long-term use associated with motor fluctuations and dyskinesias.
Dopamine agonists	Pramipexole, ropinirole, rotigotine (patch)	Direct stimulation of dopamine D2/D3 receptors.	Used as monotherapy in early PD or adjunct in advanced disease; risk of impulse control disorders.
MAO-B inhibitors	Rasagiline, selegiline, safinamide	Inhibit MAO-B, reducing dopamine breakdown; safinamide also modulates glutamate release.	Mild symptomatic benefit; adjunct to levodopa; no proven disease-modifying effect.
COMT inhibitors	Entacapone, opicapone	Inhibit COMT, prolonging levodopa plasma half-life.	Reduce OFF time when used with levodopa.
NMDA antagonist/antidyskinetic	Amantadine	Blocks NMDA receptors and enhances dopamine release.	Used for levodopa-induced dyskinesias; modest benefit.
Anticholinergic agents	Trihexyphenidyl, benztropine	Muscarinic receptor antagonism restores dopamine–acetylcholine balance.	Restricted to younger patients; avoided in the elderly due to cognitive adverse effects.
Non-motor symptom management	SSRIs/SNRIs, rivastigmine, melatonin, midodrine	Target mood, cognition, sleep, and autonomic dysfunction.	Essential for comprehensive PD management.
Non-pharmacological therapy	Physiotherapy, speech therapy, nutrition counseling	Motor rehabilitation and supportive care strategies.	Recommended at all disease stages.
Advanced therapies	DBS (STN/GPi), LCIG, apomorphine pump	Continuous dopaminergic stimulation or neuromodulation.	For advanced PD with refractory motor complications.
Non-recommended adjuncts	Inosine, coenzyme Q10	Antioxidant and mitochondrial support mechanisms.	Failed to demonstrate disease-modifying benefit in clinical trials.

**Table 2:** Overview of Parkinson’s disease-related patents.

S.no.	Title	Outcome/Remark	Patent Application No	Reference
1	Recombinant Laminin-521	Recombinant host cells express laminin-521 chains and secrete recombinant laminin-521 with a pharmaceutically acceptable carrier. The technology provides defined, xeno-free matrix conditions that enhance reproducible iPSC generation. Cell attachment efficiency reaches ~87% with ~85% spreading, generating 400 μm cell aggregates. This eliminates batch variance and contamination risks associated with undefined matrices.	US20120156254A1	[102]
2	Treatment of Parkinson’s disease	PD treatment with safinamide, safinamide derivatives, or MAO-B inhibitors in combination with levodopa/PDI or dopamine agonists. Safinamide shows a dual mechanism: selective MAO-B inhibition and glutamatergic modulation via sodium/calcium channel blockade. Clinical trials demonstrate improved motor functions and non-motor symptoms with a better safety profile than entacapone.	US8283380B2	[103]
3	New therapeutic methods for the treatment of Parkinson’s disease	Developed a sustained release formulation that could be used as a prophylactic for individuals who are susceptible to Parkinsonism. Early administration shows neuroprotective properties, reducing L-DOPA-induced dyskinesia severity. The formulation protects striatal dopamine release/reuptake and increases tyrosine hydroxylase expression. Represents shift from symptomatic to disease-modifying treatment.	JP6727259B2	[104]
4	Pharmaceutical compositions and oral dosage forms of a levodopa prodrug and methods of use	The developed formulation enhanced the bioavailability of levodopa and minimized peripheral side effects with stable plasma concentration. Prodrug approach addresses poor levodopa bioavailability (10–30%) and physicochemical limitations. Various strategies, including ester, amide, and carrier-mediated approaches, improve blood-brain barrier penetration. Overcomes degradation susceptibility and inter-patient variability.	US8435562B2	[105]
5	Subcutaneously administered treatments for advanced Parkinson’s disease	This invention disclosure showed that administering a pharmaceutical molecule via the subcutaneous route was found safe, sustained, and effective for treating PD. Bypasses irregular intestinal absorption issues. Continuous infusion increases “on” time (+3.8 h) and reduces “off” time (−3.5 h). Morning akinesia decreased from 77.7% to 27.8% after 52 weeks. Faster onset than sublingual routes (7 vs. 31 min).	US20240100072A1	[106]
6	Coenzyme Q10 solubilizing composition and method for preparing the same	Coenzyme Q10 encapsulated by micelles with glycyrrhizic acid, bile acid, and unsaturated fatty acid to improve water solubility. Addresses CoQ10′s poor aqueous solubility limitations. Micellar formulation enhances absorption and tissue distribution, increasing target site concentrations. Represents advancement in lipophilic compound delivery for neurodegenerative applications.	US20190133969A1	[107]
7	Pharmaceutical compositions and uses thereof in treating Parkinson’s disease	Developed formulation suppressed PD symptoms and significantly reversed the adverse events of the brain induced by MPTP. Contains novel NLRP3 inhibitors reducing neuroinflammation in PD pathogenesis. Protects dopaminergic neurons from degeneration and promotes recovery in MPTP-affected areas. Addresses both symptomatic relief and neuroprotective mechanisms simultaneously.	WO2022052016A1	[108]
8	Treatment regimens for Parkinson’s disease	The combination of levodopa and opicapone for treating motor fluctuations in Parkinson’s patients. Opicapone is a once-daily COMT inhibitor increasing levodopa bioavailability. Reduces “off” time by ~60 min daily without increasing troublesome dyskinesias. The OPTIPARK study showed 71.3% patient improvement after 3 months. No laboratory monitoring is required, unlike other COMT inhibitors.	WO2024039256A1	[109]

## Data Availability

No new data were created or analyzed in this study.
